# Protocol and preregistration for the CODEC project: measuring, modelling and mechanistically understanding the nature of cognitive variability in early childhood

**DOI:** 10.1186/s40359-024-01904-5

**Published:** 2024-07-26

**Authors:** Ilse E. J. I. Coolen, Jordy van Langen, Sophie Hofman, Fréderique E. van Aagten, Jessica V. Schaaf, Lea Michel, Michael Aristodemou, Nicholas Judd, Aran T. B. van Hout, Emma Meeussen, Rogier A. Kievit

**Affiliations:** Donders Institute for Brain, Cognition and Behavior, Radboud University Medical Center, Trigon Building, Kapittelweg 29, Nijmegen, 6525 EN the Netherlands

**Keywords:** Intra-individual variability, Cognitive development, Fluctuations, DSEM, Longitudinal

## Abstract

**Background:**

Children’s cognitive performance fluctuates across multiple timescales. However, fluctuations have often been neglected in favour of research into average cognitive performance, limiting the unique insights into cognitive abilities and development that cognitive variability may afford. Preliminary evidence suggests that greater variability is associated with increased symptoms of neurodevelopmental disorders, and differences in behavioural and neural functioning. The relative dearth of empirical work on variability, historically limited due to a lack of suitable data and quantitative methodology, has left crucial questions unanswered, which the CODEC (COgnitive Dynamics in Early Childhood) study aims to address.

**Method:**

The CODEC cohort is an accelerated 3-year longitudinal study which encompasses 600 7-to-10-year-old children. Each year includes a ‘burst’ week (3 times per day, 5 days per week) of cognitive measurements on five cognitive domains (reasoning, working memory, processing speed, vocabulary, exploration), conducted both in classrooms and at home through experience sampling assessments. We also measure academic outcomes and external factors hypothesised to predict cognitive variability, including sleep, mood, motivation and background noise. A subset of 200 children (CODEC-MRI) are invited for two deep phenotyping sessions (in year 1 and year 3 of the study), including structural and functional magnetic resonance imaging, eye-tracking, parental measurements and questionnaire-based demographic and psychosocial measures. We will quantify developmental differences and changes in variability using Dynamic Structural Equation Modelling, allowing us to simultaneously capture variability and the multilevel structure of trials nested in sessions, days, children and classrooms.

**Discussion:**

CODEC’s unique design allows us to measure variability across a range of different cognitive domains, ages, and temporal resolutions. The deep-phenotyping arm allows us to test hypotheses concerning variability, including the role of mind wandering, strategy exploration, mood, sleep, and brain structure. Due to CODEC’s longitudinal nature, we are able to quantify which measures of variability at baseline predict long-term outcomes. In summary, the CODEC study is a unique longitudinal study combining experience sampling, an accelerated longitudinal ‘burst’ design, deep phenotyping, and cutting-edge statistical methodologies to better understand the nature, causes, and consequences of cognitive variability in children.

**Trial registration:**

ClinicalTrials.gov - NCT06330090

## Background

Cognitive ability, measured through standardised tests, provides a highly predictive measure of lifespan outcomes including academic achievement, job success, and mental and physical health [1–3]. However, these cognitive summary measures omit a crucial aspect of cognitive performance: its short-term relatively reversible variability [4]. Our definition of cognitive variability entails fluctuations in performance metrics (e.g., accuracy, response time) within the same person across measurement occasions (trials, sessions, or even days, weeks, seasons, and years), separated from trends (e.g., systematic improvement or worsening over time). Although individual differences on tasks such as reasoning, working memory, and vocabulary have been extensively studied [1, 2] and shown to reliably predict outcomes of interest [5, 6], even such highly reliable measures may show non-trivial fluctuation within an individual over time [7]. These fluctuations have largely been ignored and (implicitly) treated as measurement error to be adjusted for [1, 8].

Cognitive variability remains understudied despite a long history highlighting its (potential) importance. As early as 1932, Woodrow [9] suggested assessing ‘quotidian variability’, a ratio of the within-day versus between-day differences, as an informative metric regarding the magnitude of day-to-day differences, and Hull [10] suggested in his chapter ‘behavioural oscillation’ that variability (or rather ‘inconsistency’) is one of the ‘chief molar distinctions between organisms and inorganic machines’ (p. 304). Cattell [11] referred to overlooking of variability as a ‘moral failure’, because a neglect of variability may lead to various potential errors in, for instance, high stakes assessments.

After a long dearth of empirical work on variability, interest in variability was reignited with studies showing cognitive variability on different timescales, including trials, hours, days and even seasons [12–15]. This variability seems to be more pronounced during periods of rapid cognitive growth or decline, such as in childhood and old age [16–18]. Moreover, variability is a potent cognitive markers of neurodevelopmental disorders such as attention-deficit/hyperactivity disorder (ADHD; [19–21]) and symptoms may even be alleviated using pharmacological interventions targeting cognitive variability [21], emphasising the importance of variability as an etiologically pertinent correlate of neurodevelopmental disorders.

Despite evidence for variability on different timescales, its predictive value for neurodevelopmental disorders, and potential pharmacological interventions, much about cognitive variability remains unknown. Open questions include, but are not limited to, how cognitive variability differs between individuals and across different timescales, its behavioural and neural determinants, and the long-term (positive or negative) consequences. Better understanding of these processes is crucial for both fundamental and translational goals. For instance, highly variable individuals may have a greater chance of under- or over-performing during high-stakes assessment. A better understanding of variability will allow us to better tailor environments in which cognitive performance has strict lower bounds for safety (e.g., pilots or surgeons, [17]), as already implemented in various legal mandates such as those imposing upper limits for daily driving time (e.g., 9 hours for truck drivers in the EU, [22]).

**Fig. 1:**
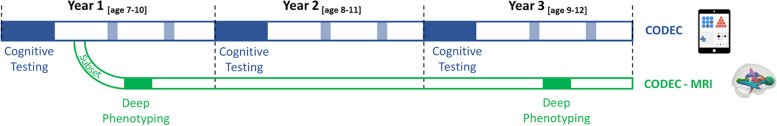
The CODEC design combines high intensity experience sampling ('burst' sessions in blue and potential additional testing sessions in light blue) using mobile tablet measurements in classroom schools (or at home). A 'burst' session corresponds to a week during which children perform cognitive testing up to three times a day. A subset of participants will also undergo deep phenotyping (MRI scanning during the first and last year of the study) additionally to the cognitive testing in schools (or at home)

This chance of under- or over-performing on high-stakes assessments demonstrates the fundamental scientific as well as translational importance of better understanding variability. The CODEC project is designed to overcome a range of previous shortcomings, offering new insight into cognitive variability. More specifically, the CODEC study focuses on several core questions, including (1) how does cognitive variability differ between children and across different developmental and temporal scales; (2) what environmental, psychological, and neural mechanisms underlie cognitive variability, and (3) what are the long-term outcomes associated with differences in cognitive variability in childhood? To address these questions, the CODEC study consists of a longitudinal ‘burst’ design using game-like (visually engaging) versions of five cognitive domains (i.e., working memory, reasoning, processing speed, vocabulary, and exploration) measured across a range of temporal resolutions. To achieve this goal, for a period of 3 years, children are tested on these five domains throughout the year. Children will be tested for up to three times a day for one week per year (the ‘burst’ week) and with up to two occasional testing sessions (one single measurement) throughout the rest of the year (see Fig. 1). Most studies on variability have focused on single-occasion measurements (quantifying variability across a sequence of trials). Those that have included a longitudinal focus have tended to focus on relatively short-term periods (e.g., 30 days; [16]). The longitudinal design of the CODEC study allows us to investigate both a wider range of temporal resolutions (days, weeks, months) as well as the long(er)-term outcomes (years) associated with differences in cognitive variability.

The CODEC study will focus on three main topics: (1) the structure of individual differences in variability, (2) potential causes of these individual differences, and (3) developmental trajectories and long-term outcomes associated with individual differences in variability.

### Individual differences in cognitive variability and their specificity

Our first focus is on the extent to which individuals fluctuate in cognitive performance and how these fluctuations differ between individuals. This relatively simple question allows us to establish first and foremost the nature of systematic and replicable individual differences in cognitive fluctuations, and to what extent these fluctuations provide valuable information about individuals.

We investigate such individual differences in cognitive variability across different timescales (trials, sessions, days, seasons and years) and cognitive tasks (visuospatial working memory, reasoning, processing speed, vocabulary, and exploration). In line with previous studies, we suspect there is valuable information in fluctuations across different timescales, including day-to-day and day-of-week dynamics [23], and seasonal trends [24]. Crucially, recent evidence [16, 25, 26] suggests that individual differences at different temporal resolutions (e.g., trials versus days) are at most weakly correlated, suggesting distinct underlying mechanisms. Moreover, we also examine the degree of similarity across tasks – In other words, if children are relatively variable on one task, are they also likely to be more variable on others? Our previous findings suggest trial-to-trial variability is likely correlated across tasks, but much more weakly so than mean performance [7].

### Predictors and potential causes of cognitive variability

To better understand which variables explain individual differences in variability, we will measure a range of environmental (i.e., socio-economic situation, noise), behavioural (i.e., psychological and cognitive factors) and neural factors hypothesised to underlie or be associated with greater or lesser variability within and between persons. Identifying which environmental, behavioural, and neural determinants influence within-person cognitive fluctuations is crucial to better understand the underlying mechanisms governing individual variability. It is highly likely that no single behavioural or environmental factor will fully explain cognitive variability - for this reason, we incorporate a rich set of potential predictors to provide a more comprehensive understanding of within-person cognitive variability.

#### Environmental factors

##### Socio-economic and hereditary background

Socio-economic status (SES) has often been documented as being associated with children’s cognitive performance (e.g., [27]). An indirect link between SES and variability in cognitive performance has also been suggested. Specifically, Gearin et al. [28] showed that SES could predispose individuals to mind wandering patterns, which in turn is known to relate to cognitive performance [28] and variability [29–31]. In light of these findings, the socio-economic environment of a family could impact a child’s cognitive variability. Beyond these familial environments, hereditary factors might further (at least in part) explain variability [32]. For this reason, a subset of parents will perform the same cognitive tasks as their children, allowing us to quantify the within-family similarities above and beyond other factors.

##### Noise

Environmental noise can interfere with cognitive processes, resulting in, for example, reduced attention while performing cognitive tasks [33], which may lead to more variability in performance. However, some research highlights how white background noise while performing a task can improve cognitive functioning, such as inhibition in children with ADHD [34, 35], potentially by reducing cognitive variability. This suggests an interesting pattern, that children with ADHD symptomatology have greater cognitive variability than children without such symptomatology, but that unlike neurotypical controls, they may benefit from (some types of) noise. This could mean that some children require silent surroundings to perform optimally on cognitive tasks (and thus would demonstrate more cognitive fluctuations in noisy environments), while for others the opposite holds.

#### Behavioural factors

In addition to environmental factors, several psychological (i.e., sleep, mood, motivation, environmental sensitivity) and cognitive factors (i.e., cognitive functioning and cognitive strategies) are likely to be associated with cognitive variability in children.

##### Psychological factors

Other than cognitive performance, factors such as mood, sleep quality, and motivation also fluctuate within individuals in ways that could be summarised as ‘bad’ (i.e., sleep deprivation, negative mood or low motivation) and ‘good’ days, with plausible consequences for cognitive performance. Furthermore, the impact that those factors have on cognitive performance is likely to differ between individuals, with some being more sensitive than others.

**Sleep**

It is well established that sleep deprivation has pronounced effects on cognitive performance [36]. More specifically, sleep deprivation has been shown to affect trial-to-trial variability on a one-choice decision task [37], suggesting that poorer sleep at night is a contributing factor to higher cognitive variability during the day. Fluctuations in sleep quality can be identified in young children and sleep quality has been positively related to cognitive performance in the morning, but not necessarily later in the day [38]. Furthermore, sleep quality becomes increasingly important for daily performance as children develop between the ages of 6 to 18 years [39].

**Mood**

In intensive longitudinal data, mood is robustly found to be unstable over time [40], and fluctuations in mood have been associated with performance fluctuations such as in job [41] and cognitive performance [42–44]. In fact, within psychopathology there has been a longer tradition of treating the variability or volatility of mood symptoms as a key and clinically relevant feature above and beyond the absolute levels (e.g., [45]). Given this constellation of previous findings, we hypothesise that day-to-day differences in mood will likely be related to day-to-day differences in cognitive performance. Between-subject positive associations have been found between affect and working memory performance [46], but whether this association also manifests within-persons across time (and tasks) is yet to be determined.

**Motivation**

Previous work suggests that a greater interest in a task relates to higher task performance [46]. The contribution of motivation in within-subject differences can be examined by comparing and contrasting the predictive role of (subjective) interest in each task. If motivation or enjoyment play a key role in governing variability, we would expect that a subjective assessment of ‘most to least fun’ task would correspond to a similar, scaled ordering of variability. Previous findings found that motivation was task independent [47]. However, whether their enjoyment for a task impacts their fluctuations in the task performance is poorly understood, and will be examined in this study.

**Psychological profiles**

To better understand individual differences in cognitive variability, we have to consider not only continuous variation in variability parameters, but the possibility that there exist latent subgroups characterised by qualitatively distinct profiles. A previous study by Neubauer et al. [43] provided evidence that such latent subgroups are plausible. The authors observed four subgroups of individuals characterised by more or less pronounced links between mood and cognitive variability (i.e., low mood leading to higher variability, high mood leading to higher variability, mood having little effect on variability, and a group where both low and high mood lead to higher variability). Indeed, the varying effect of factors such as mood, sleep, motivation, and surroundings (e.g., noise) on cognitive variability between individuals can be investigated by means of identifying different profiles (e.g., subgroups of individuals with a high impact of mood, sleep, motivation or environmental sensitivity on variability compared to a subgroup with low impact of these factors or mixed interactions between them). Both continuous differences and typological differences may prove informative about the mechanisms and outcomes of variability phenotypes. Given previous evidence and our larger sample size, we expect to identify different profiles of individuals in whom mood, sleep, motivation, and environmental sensitivity would have varying effects on variability.

##### Cognitive factors

**Cognitive functioning**

Inattention has consistently been identified as a strong and specific predictor of cognitive variability, specifically variability in response times. For instance, Aristodemou et al. [19] found that higher inattention, but not hyperactivity, showed higher cognitive variability, with similar patterns observed in other studies [48, 49]. Two studies have also shown that a reduction in attentiveness as a function of time on task leads to an increase in response time variability [19, 30]. Lastly, longitudinal studies show that inattention symptoms are associated with greater cognitive variability at follow-up [50, 51].

Similar patterns are observed for the adjacent but distinct construct of mind wandering. Mind wandering reflects spontaneous changes in attention (lapses), which are related to variability (e.g., [52]), and may cause variability in cognitive performance [39]. For instance, Unsworth and Robinson [30] demonstrated that on occasions when individuals reported mind wandering, they had lower (relative) performance on working memory tasks. Conversely, individuals with lower (baseline) working memory performance, reported more frequent mind wandering. Mind wandering could be a basis for attentional lapses causing more cognitive variability, but mind wandering in turn can also be triggered by poor executive control in tasks where sustained attention is required [53].

On the other hand, it may be plausible that mind wandering plays a positive role in (long term) cognitive performance, supporting the generation and exploration of novel ideas in the creative process [54]. Mind wandering could facilitate the process of finding more remote associations to the topic, resulting in the generation of more original ideas or solutions in problem-solving [55]. In the CODEC study we further investigate such relations between mind wandering and cognitive variability.

**Cognitive strategies**

Another cognitive factor associated with variability is the use of cognitive strategies. For instance, individuals that use a single strategy to solve a task will likely be less variable than individuals that try out multiple strategies [56]. Individuals who try out more distinct strategies are therefore more likely to show greater variability, but in a manner that could yield more rapid long-term growth as the individual is more likely to discover the task- or person-specific optimal strategy [57]. To investigate this potential positive predictive value of variability, we therefore include three tasks (an exploration task, a spatial working memory task and a fluid reasoning task) in which known strategies have been identified, and can be investigated using computational modelling.

##### Exploration

In many situations in daily life, there exist a wide range of options – for instance, whom to interact with, what food to eat, and which activities to engage in. In many such settings, individuals must decide between exploring unknown (but potentially highly positive) options, and exploiting options with known properties. Solving this exploration-exploitation dilemma requires balancing the costs and benefits of exploration (sometimes called ‘choice variability’) based on the requirements of the environment. In the CODEC study, we use computational modelling to investigate how well children adapt their level of exploration to the environment, how explore and exploit tendencies are associated with variability, and how these processes change with age. Two recent studies have investigated these questions using cross-sectional designs [58, 59],we here do so using a longitudinal design. We are specifically interested in the predictive value of adaptive exploration for later outcomes, as previous cross-sectional studies have linked overexploration to symptoms of ADHD known to covary with variability [60–62].

**Strategy shifting**

Individuals may not only adapt the degree of exploration to the requirements of the environment, but also the type of cognitive strategy they use. Specifically, evidence suggests that variability may reflect that some individuals try out different strategies to solve a cognitive task [57, 63–65]. Evidence in children suggests that such strategy exploration is prevalent and differs between individuals [66]. For instance, in fluid reasoning tasks, individuals may use either constructive matching, response elimination, or a hybrid combination of the two strategies [66]. Here, we test whether switching between these different cognitive strategies across trials drives increased variability in a fluid reasoning task. If so, this may reflect a specific subtype of variability that may be adaptive in the long term, because it leads to optimised strategy use over time. As such, strategy exploration and -switching could relate to more rapid long-term growth despite worse short-term performance. Notably, differences in task demands (e.g., time limits) are known to lead to differences in strategy use [66, 67]. We will leverage this finding, and have children perform two versions of a fluid reasoning task in the scanner to help us disentangle distinct strategies switching. If trial-to-trial fluctuations reflect systematic shifts between strategies, then different trial types should be associated with clearly separable neural networks. If, in contrast, fluctuations merely reflect inattention, then the neural networks underlying different responses should differ in degree (extent and magnitude) not in kind (spatial location).

#### Neural factors

Other predictors of cognitive variability we consider are brain structure and function. With respect to structure, it has been shown that individual differences in cognitive variability are linked to differences in white matter microstructure [49, 68] and that a reduction in cognitive variability over time relates to the development of white matter microstructure [69]. Specifically, the neural noise hypothesis states that myelination of axons leads to less noisy transmission of brain signals, and ultimately less behavioural variability [70].

Apart from brain structure, individual differences in cognitive variability have also been linked to variability in brain function (i.e., neural variability; [17, 71–74]. One interpretation of neural variability is that it reflects neural noise which affects our ability to parse weak and ambiguous signals, directly *causing* cognitive variability [75, 76]. In contrast, when using functional magnetic resonance imaging (fMRI), neural variability, measured through the blood oxygen level-dependent (BOLD) signal (with higher BOLD signal indexing lower neural noise), has been associated with better cognitive performance, as well as lower variability in performance [77]. A different interpretation of fMRI-based BOLD variability is that it reflects the dynamic range of a person’s brain, with higher variability reflecting the ability to occupy a greater range of states (e.g., alert, on-task, relaxed) and readily respond to changing environments. This interpretation is supported by studies linking greater functional connectivity with higher neural variability to meet increasing task complexity [74]. Neural variability may index both neural noise and the ability to switch between states, as these are features that can be modulated by the excitation-inhibition balance, which refers to the balance between excitatory (promoting neural activity) and inhibitory (reducing neural activity) neural signals [78].

Apart from global functional connectivity, the number of functional networks that are recruited provide another source of information about the possible cause of cognitive variability. Empirical evidence shows that a greater range of cognitive strategies during behavioural tasks has been associated with the recruitment of more distinct functional networks [79]. We investigate the relation between structural brain measures and variability in all tasks. We further investigate functional brain measures and eye-tracking measures and relate this to variability in the fluid reasoning task to disentangle different cognitive strategies used in the task with time constraints compared to the task without time constraints.

### Developmental trajectories of cognitive variability

A key question that the CODEC study seeks to address is to better understand the developmental trajectories of cognitive variability, and whether the shape and speed of this development differs between children. Early results consistently suggest that within-person trial-to-trial variability is present in people across the entire lifespan, both at the behavioural and cognitive level [64]. In childhood, cognitive variability has been demonstrated in infancy [80], 18-to-35 month olds [81], preschool children [56, 82], and older children [83–85]. Longitudinal studies in childhood are sparse, but it has been suggested that children’s variability decreases across childhood from the age of 6 years to adolescence [17], potentially reflecting their relation to mean performance and myelination. Moreover, a recent study by Cubillo et al. [86] found that variability on a working memory task decreased with training over time and that this variability was a predictor of academic outcomes 6–12 months later.

Later in life, variability increases again as demonstrated in cross-sectional findings, which is in association with observed lower cognitive performance in older age [17, 87]. Longitudinally, cognitive variability increases linearly from early adulthood into late adulthood and predicts cognitive decline [88–90]. Together, these studies suggest a U-shaped pattern across the lifespan, with high variability in early life as well as old age [90–92].

### Long-term predictive value of cognitive variability

Besides investigating variability at each measurement and its development across measurements, we are also interested in the predictive value of variability for later outcomes. For instance, cognitive variability has been shown to forecast between-subject differences in important developmental outcomes such as academic achievement [86], and children’s neurodevelopmental symptoms such as ADHD and Autism Spectrum Disorder (ASD) [93–95]. This suggests that early variability could be used as a marker for the detection of later neurodevelopmental disorders, but longitudinal data are scarce. Another example of the predictive value of variability for later outcomes is based on children’s developmental changes and learning processes with regards to exploring and refining cognitive strategies. As children grow older, their strategy-related variability might diminish as the creation of novel neural connections decreases [96]. Thus, the degree of children’s exploration, their cognitive variability, and their white-matter microstructure potentially relate. This association between changes in neural and cognitive variability over time and across various cognitive domains has not been examined to date. While higher variability has been related negatively to cognitive performance [26], in tasks that require exploration, cognitive variability might actually improve performance because it allows children to try out different strategies. This behavioural variability could manifest as greater neural variability and non-overlapping neural networks during tasks. This means that in some tasks, there could be a positive relation between neural and cognitive variability.

We will test the predictive value of variability for academic achievement, cognitive growth (i.e., slopes in mean performance), neurodevelopmental disorder symptomatology (e.g., inattention, cognitive control), and additionally for cognitive performance (on all tested domains) and neural outcomes.

## Methods and design

### Study design

#### CODEC

The CODEC study is an accelerated longitudinal observational cohort study involving 600 children aged between 7 and 10 years at the first measurement, who are followed for a duration of 3 years. Participants will be included from the summer of 2024 to December 2027, or longer if necessary to test all children a final time after 3 years. The study consists of two arms: behaviour only, and behaviour + imaging. Children take part in the behavioural study either in classrooms or on an individual basis. They are tested once a year for a ‘burst’ measurement (2–3 times a day for 5 days a week) and up to 2 additional brief (1 testing session of 25 min) measurements a year (see Fig. 1). Frequent sampling is required to separate different temporal resolutions (e.g., trial-to-trial, occasion-to -occasion, day-to-day), ensure appropriate power, and separate developmental effects from retest effects. The benefits of school-based assessment are threefold: First, the classroom is a naturalistically relevant setting to understand cognitive testing. Second, the centralised assessment occasions will maximise cognitive variability compared to children being able to select the time and place when they wish to perform the tests. Third, the measurement of environmental noise (see below) in the classroom reflects a translationally relevant challenge of silence in educational settings. On the other hand, the recruitment through the individual route will allow us to compare the magnitude of individual differences in variability to those recruited in the classroom setting. We hypothesise that the self-paced nature of testing at home may allow participants to avoid ‘troughs’ of fatigue or poor performance.

#### CODEC-MRI

A subset of ~200 participants will also take part in a neuroimaging arm, which consists of two MRI sessions at the Donders Institute for Brain, Cognition and Behaviour (in the first year of testing and approximately 3 years later). This enriched arm of the study, including a set of standardised questionnaires, will allow us to determine the role of brain structure and function in supporting cognitive dynamics, capture a richer picture of the psychological phenotypes associated with variability, and enables us to determine mechanistic underpinnings of variability.

### Study population

Six hundred children are recruited from schools and on an individual basis to take part in the behavioural testing. Every child who participates in the behavioural arm is invited to participate in the imaging arm (CODEC-MRI) at the Donders Institute. Based on previous experiences in similar studies, we expect approximately 1/3 of children will also enrol in the imaging arm, for a sample size of 200. Parents or caretakers of this subgroup are also asked to respond to a set of questionnaires and take part in the behavioural tasks.

In order to be eligible to participate in the behavioural arm of this study, a subject must meet all of the following criteria:

- Between the ages of 7 and 10 years at the moment of the first assessment.^1^
- The participant is able to understand the instructions of the behavioural tasks given in the Dutch language.
- Parents/guardians have provided written informed consent.

In order to be eligible to participate in the imaging arm of this study (CODEC-MRI), a subject must meet all of the following criteria:

- Between the ages of 8 and 10 years at the moment of the first MRI assessment.
- The participant is able to understand the instructions of the behavioural tasks given in the Dutch language.
- No history of neurological or psychiatric illness.
- No history of using psychotropic medications.
- No metal parts that cannot be removed, are present in or on the upper body, e.g., plates, screws, aneurysm clips, metal splinters, piercings or medical plasters (exception: dental fillings, crowns, a metal wire behind the teeth, tattoos).
- Body does not contain metal fragments, in particular in the eye, e.g., caused by injuries.
- No history of brain surgery.
- No active implant(s) (e.g., pacemaker, neurostimulator, insulin pump, ossicle prosthesis).
- Not using a medical plaster that cannot or may not be taken off.
- Parents/guardians have provided written informed consent.

### Recruitment and enrolment

There are two initial recruitment routes. Our core recruitment strategy will be through schools in the Netherlands, mainly in and around Nijmegen. The other recruitment is at the individual (non-school based) level, discussed later.

During school recruitment, schools are contacted and receive information on the study. This information is in the form of an email, an information sheet, and a link to the CODEC study website [97] including informational videos of the aims and procedure of our study. Schools can be contacted by phone to follow-up several days after receiving the information by email. Parents/guardians receive an information sheet and consent form from the participating school and are asked to sign and return the informed consent when giving consent for their child to take part in the study.

Individuals willing to take part in the study, but whose school/classroom does not or cannot participate, can be recruited on an individual basis. The parents/guardians who have expressed interest in participating receive a dedicated informed consent and information sheet either in person, by post or email, depending on their preference. Parents or guardians of participants are asked to give, or decline, consent for 1) participation in the behavioural study, and additional consent 2) to be contacted to receive more information about the imaging study, 3) for the researcher to obtain Cito scores (or equivalent academic results) through the school (only for those children recruited in classrooms), and 4) to be contacted to receive more information for other follow-up studies. Agreeing to 1 is possible without agreeing to 2, 3 and/or 4. For participants consenting to be contacted for the imaging study or follow-up studies, the preferred means of communication (phone or email) is requested, and the relevant information provided. Parents are contacted through their preferred means of communication (either through post, email or in person) and receive the information sheet and informed consent for the imaging part (if agreed upon). Parents or guardians give consent on the ‘imaging’ informed consent form to have their child take part in the imaging study (CODEC-MRI). An additional informed consent form asks parents/guardians to perform the cognitive battery tasks, identical to the tasks performed by the child in the behavioural study and/or to respond to a set of questionnaires on their child and their environment. Parental participation is not necessary for the child to enrol in CODEC-MRI. A new consent form needs to be signed for the child to take part in the second MRI scan and for the parents/guardians to take part in the second accompanied behavioural tasks and questionnaires. 

### Study procedures and assessments

#### Behavioural procedure – school recruitment

Initial ‘burst’ week - The CODEC team organises an initial visit to the schools with the relevant teachers and hands out tablets to the children. All children perform the same tasks, but no data is collected for children for whom consent was not given. Children perform the tasks in the classroom, independently but in a group setting, for 2–3 times a day for the duration of a week using the same tablet, supported by teachers in the classroom. The precise timing of the measurement occasions is determined in collaboration with the teacher, to accommodate the school’s schedule, and thus will vary between classrooms. The first testing session will be assisted by a member(s) of the CODEC team to ensure that all task demands are clear, and any logistical (e.g., login) problems can be addressed immediately. Children have the opportunity to ask questions, and tick a box prior to starting the first testing session indicating that they have understood all instructions. In each testing session, three of the five tasks (selected pseudorandomly) are played by the child for the duration of 5 min (15 min in total) to make sure that the session remains sufficiently short and exciting for the children.

Follow-up measurement occasions - Throughout each year of testing, up to two additional testing occasions can be planned in agreement with the teachers. On these occasions, the procedure is identical, but all five cognitive tasks are performed once in a single session. Two follow-up ‘burst’ weeks take place after approximately one (in the second year of the study) and 2 years (in the final year of the study), for which the procedure is identical to the initial ‘burst’ week.

#### Behavioural procedure – individual recruitment

The procedure for individual participants is similar to the participation through schools, but testing is done in a home setting. Participants at home use an individual login-code on a personal tablet or a tablet provided by us to the parent. The parent and child are provided with all necessary instructions, guidelines and information. The first testing session may be accompanied by a CODEC team member if preferred by the parent(s) or participants. Up to two yearly, shorter additional testing occasions are possible in agreement with the parents, identical to the additional testing occasions in the school recruitment, and a yearly ‘burst’ week follow-up is organised with the parents in the same way as the initial testing session.

#### Behavioural assessment

The cognitive task battery used for behavioural testing in schools is implemented on the m-Path platform [98, 99] and is performed on Samsung Galaxy Tab A8 tablets (10.5-inch TFT screens with a standard 60Hz refresh rate). Individual participants not recruited through schools can use their own tablet as long as it is compatible with m-Path. The test battery measures a series of classic cognitive tasks: working memory, reasoning, processing speed, vocabulary and exploration (more detail below). Each task yields a set of estimated phenotypic parameters including speed, accuracy, trends, autoregression and variability which will vary across measurement occasions. The participants receive verbal instructions that they will play some games and are instructed to only use one finger at a time to play all the games. Each game will allow them to gain points as a way to make the tasks more engaging for children.

##### Cognitive tasks

**Fig. 2:**
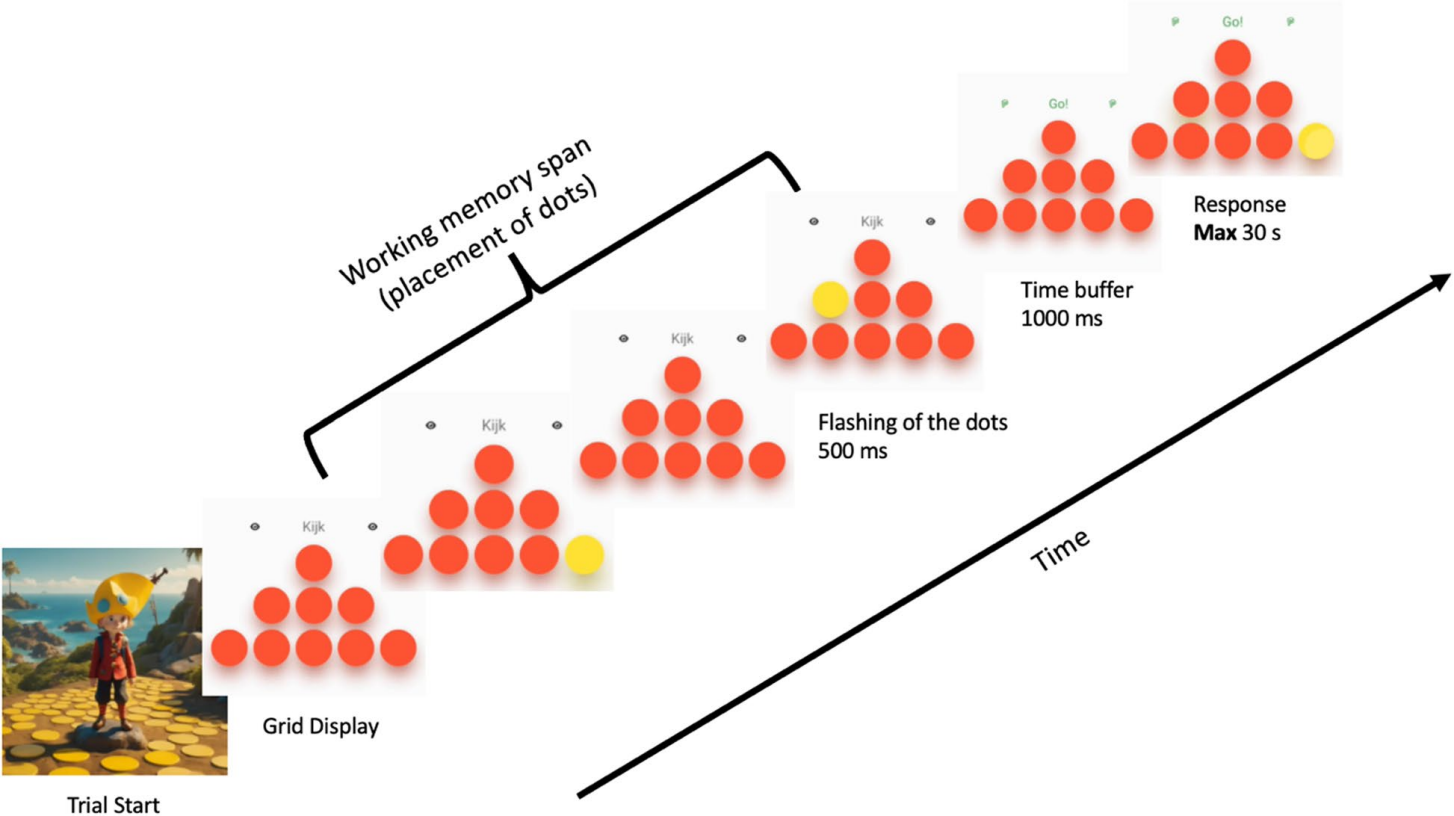
Working memory task procedure. Participants are presented with a shape of dots (e.g., triangle), after which a sequence of dots turns yellow sequentially. Participants are instructed to copy the sequence in the same order

*Working memory:* to measure visuo-spatial working memory performance, we use a task based on a Corsi-Block-Tapping task [100]. In this task, participants are instructed to recall and reproduce a sequence of flashing dots by pressing the dots in the correct order (see Fig. 2). The maximum number of correctly pressed dots is considered the upper limit of working memory span. In our task, named ‘Stippenweg’ (‘Dotted Road’), participants see a grid consisting of red dots on a white background. The dots are arranged in different shapes (e.g., circle, square) that switch across trials to increase engagement. The total number of dots in each grid varies between 9 and 16. At the start of each trial, a number of dots sequentially turn yellow for 500 ms in a pseudorandom sequence. After the last dot in the sequence, there is a 1000ms inter-stimulus interval. Then participants receive a visual and written cue instructing them to start recalling the sequence by pressing each displayed dot in the correct order. After each stimulus, participants receive feedback: trials can be completely correct, partially correct or completely incorrect. Participants gain points for accuracy - 50 points per correctly reproduced dot - and speed - up to a maximum of 100 speed points per trial. The task is adaptive to performance, starting with two dots and increasing the sequence span by one dot after four consecutive correct trials and decreasing it by one dot after four consecutive completely incorrect trials (zero dots in the correct order).

**Fig. 3:**
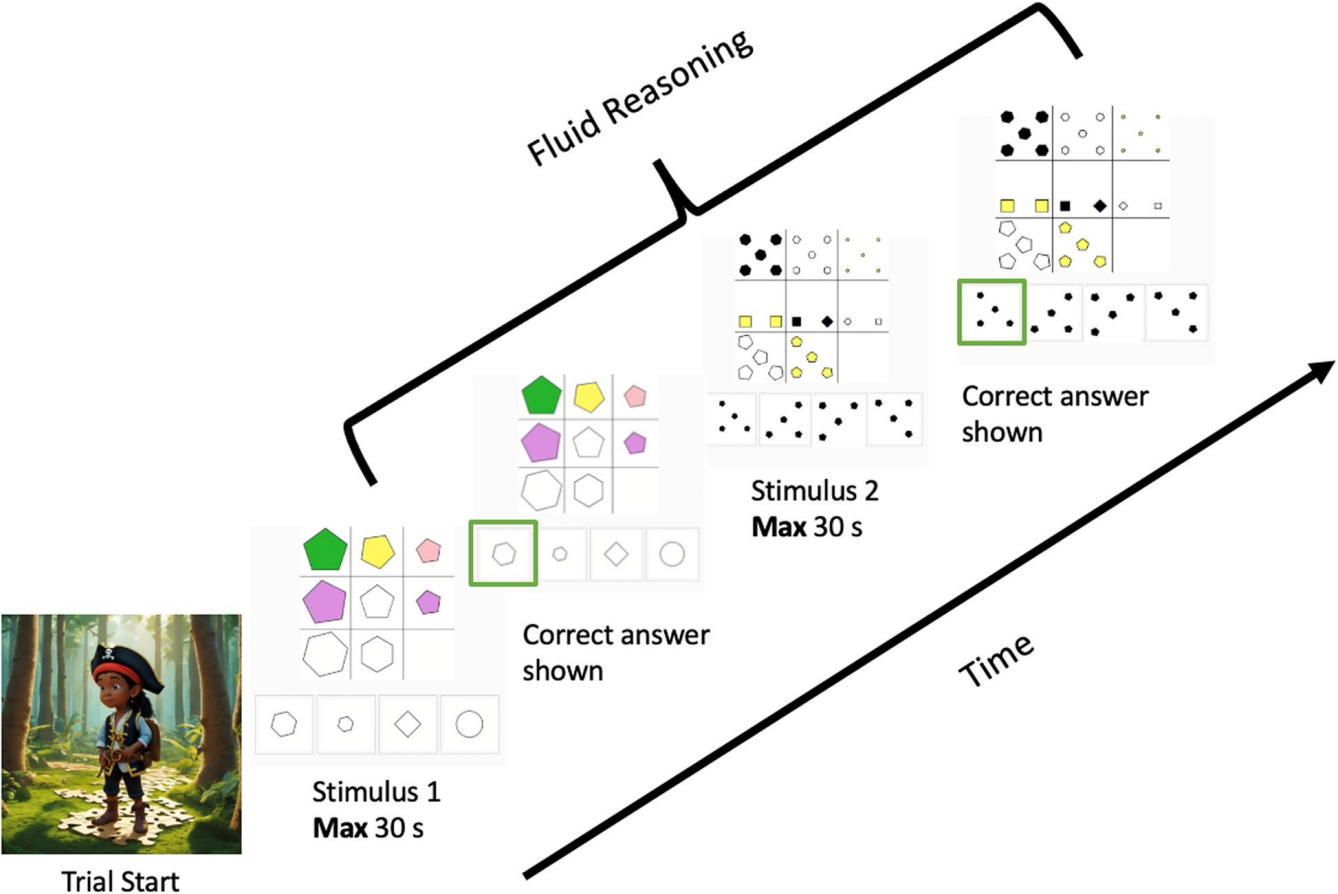
Fluid reasoning task procedure for 2 example trials. Participants are presented with a 3-by-3 grid. Each cell contains one or more elements, except the bottom right cell. Participants are instructed to decide which out of four response options fits best in the empty cell

*Fluid reasoning:* to measure fluid reasoning, we use a matrix reasoning task similar to Raven’s Progressive Matrices [101, 102] or Cattell’s Culture Fair test [103]. As existing tests are proprietary (precluding reuse by others with fewer means), and even open source alternatives, e.g. MaRs-IB, [104] have a limited number of items, we developed our own battery of items. To do so, we adapted an existing pipeline [102] and generated 3-by-3 grids with elements varying across five dimensions: shape, colour, size, number and orientation. Crucially, item difficulty is modified following the rules outlined in Carpenter et al. [101]. During the task, participants see a 3 by 3 grid from which all cells contain one or more elements, except the bottom right cell, which is left empty. Participants are asked to select, out of 4 options, which option best fits this empty cell of the grid (see Fig. 3). In the behavioural arm of the study, there is a time limit of 30 s on the response. In the deep phenotyping arm, participants perform the same task with either low time constraints (30 s to respond) or high time constraints (10 s to respond) (see CODEC-MRI: imaging procedure). After each response, the correct answer is outlined in green. After finishing all items in a block/session, participants see their total score (number of correct answers) on the screen.

**Fig. 4:**
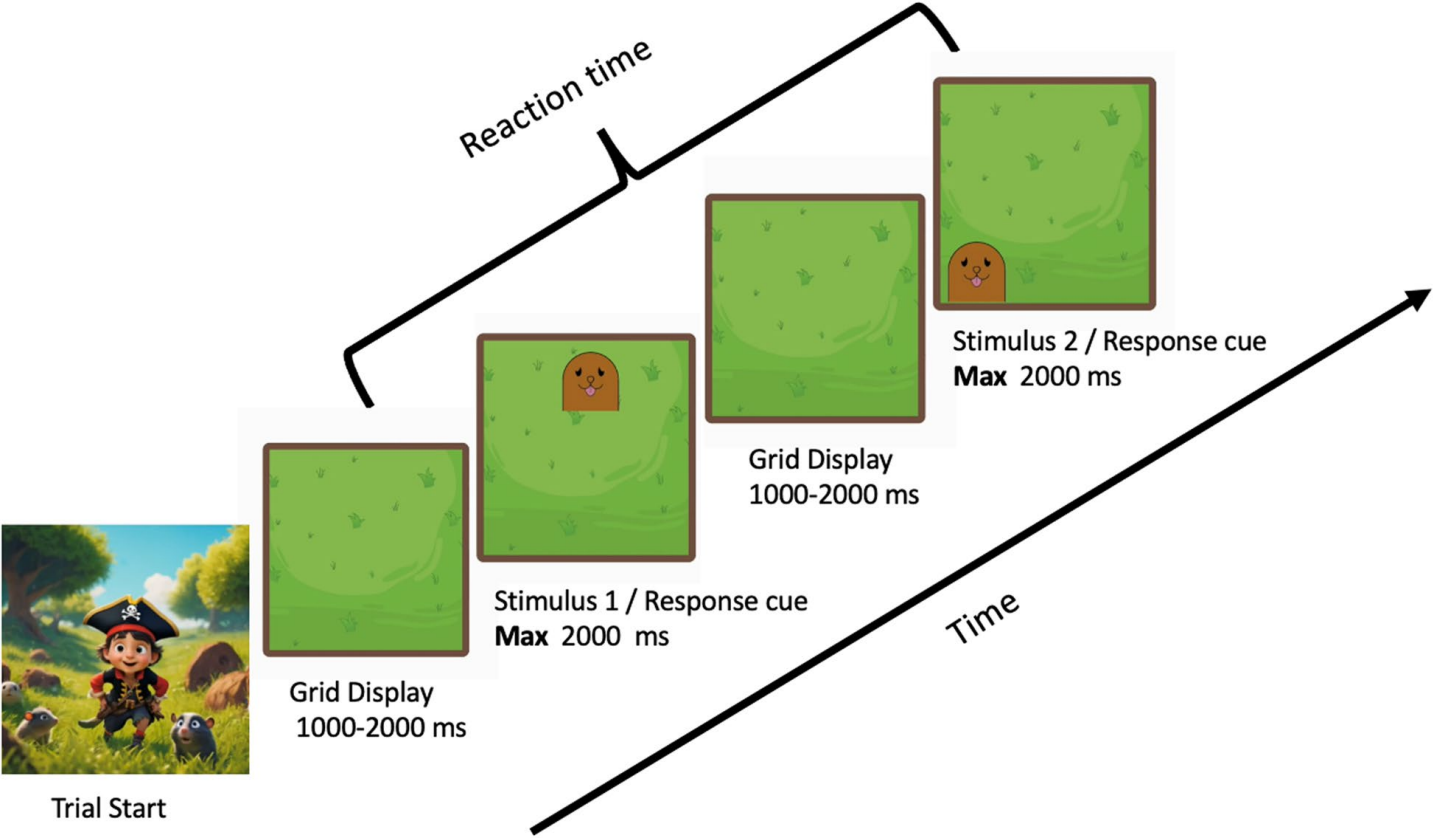
Whack-a-mole task procedure. Participants are presented with a digital grass field. Per trial, 10 mol will appear sequentially. Participants are instructed to tap the appearing mole as quickly as possible

*Processing speed:* to measure processing speed, we use a version of the classic ‘Whack-a-Mole’ game. Participants view a (digital) grass field, with a cartoon mole appearing at unpredictable locations on an invisible 3-by-3 grid (see Fig. 4). The participants’ task is to tap the moles as quickly as they can, before they disappear. Moles are presented on the screen one by one with a total of 10 mol per trial. A mole disappears once it is pressed or after 2000ms. A new mole appears at an interval selected randomly between 1000 and 2000ms. To prevent children from clicking randomly, clicking on an empty section of grass will cause a mole to disappear without gaining points.

Participants are awarded points for both accuracy and speed. Feedback on total score and speed is provided after each series of 10 moles. Besides accuracy and reaction time, data collected includes the location of the finger press, calculated on a 9-by-9 invisible grid underlying the grass field. This enables the calculation of distance between the mole and the actual finger press with greater accuracy.

**Fig. 5:**
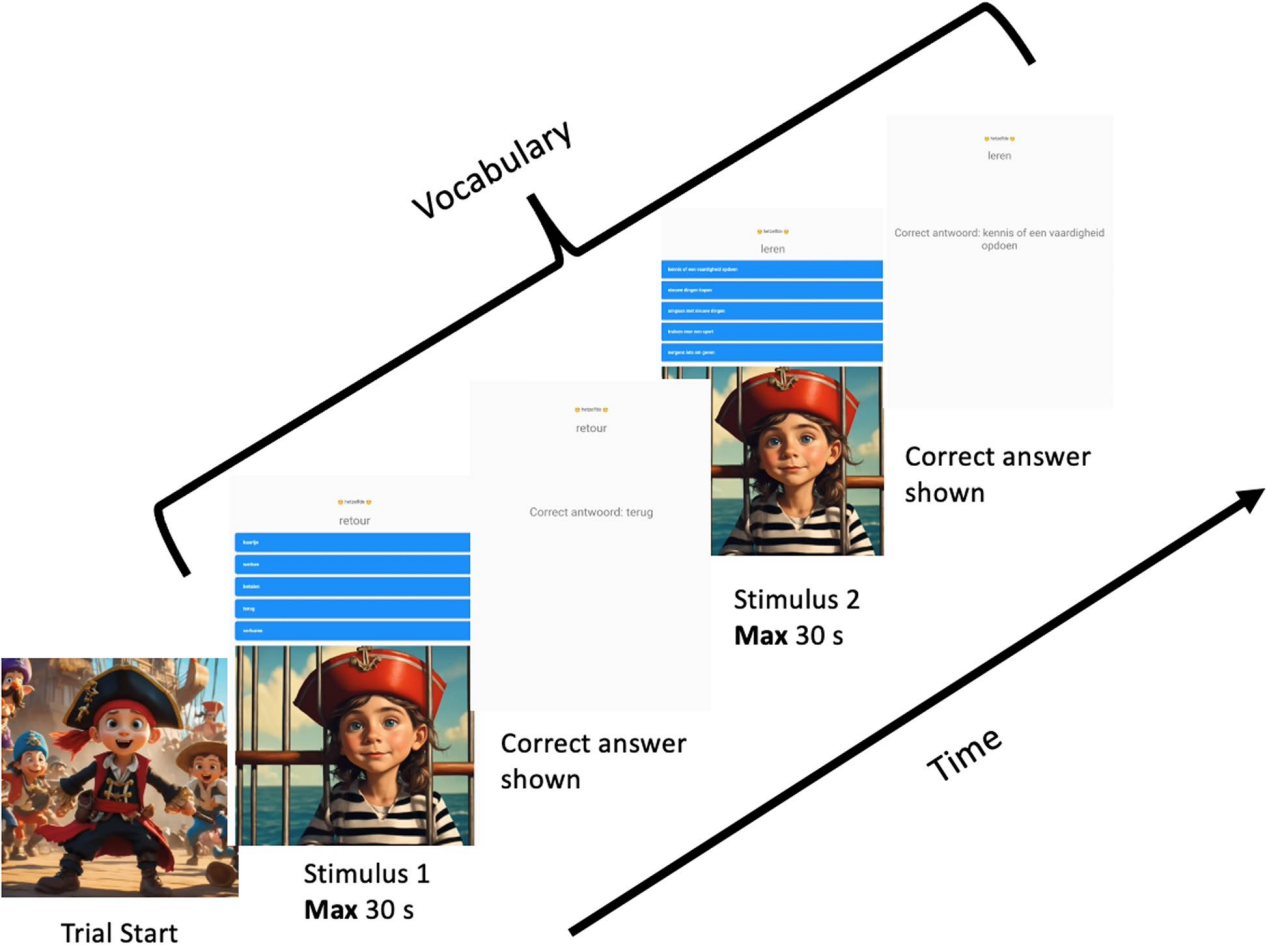
Vocabulary task procedure. Participants are presented with a word and 5 response options. In the first half of the task, participants are instructed to pick the response which best describes the target word. In the second half of the task, participants are instructed to pick the response which best fits the opposite meaning of the word

*Vocabulary:* In order to measure vocabulary, we use a multiple-choice vocabulary task (see Fig. 5) based on the online learning platform Taalzee [105, 106]. We sample a set of 2000 words and alternatives previously used and validated in Taalzee. In the first half of the task, participants see a word and are asked which of five alternatives best fits the description of the word. In the second half of the task, participants are asked which of five alternatives best fit the opposite meaning (antonym) of the word. The order of the five alternatives is randomised at each round and the correct answer is provided after each exercise. Participants receive points for accuracy and speed. There is no time limit on each trial, however, after a series of 5 answers with a reaction time under 750 ms - suggestive of guessing - the participant is instructed to slow down.

**Fig. 6:**
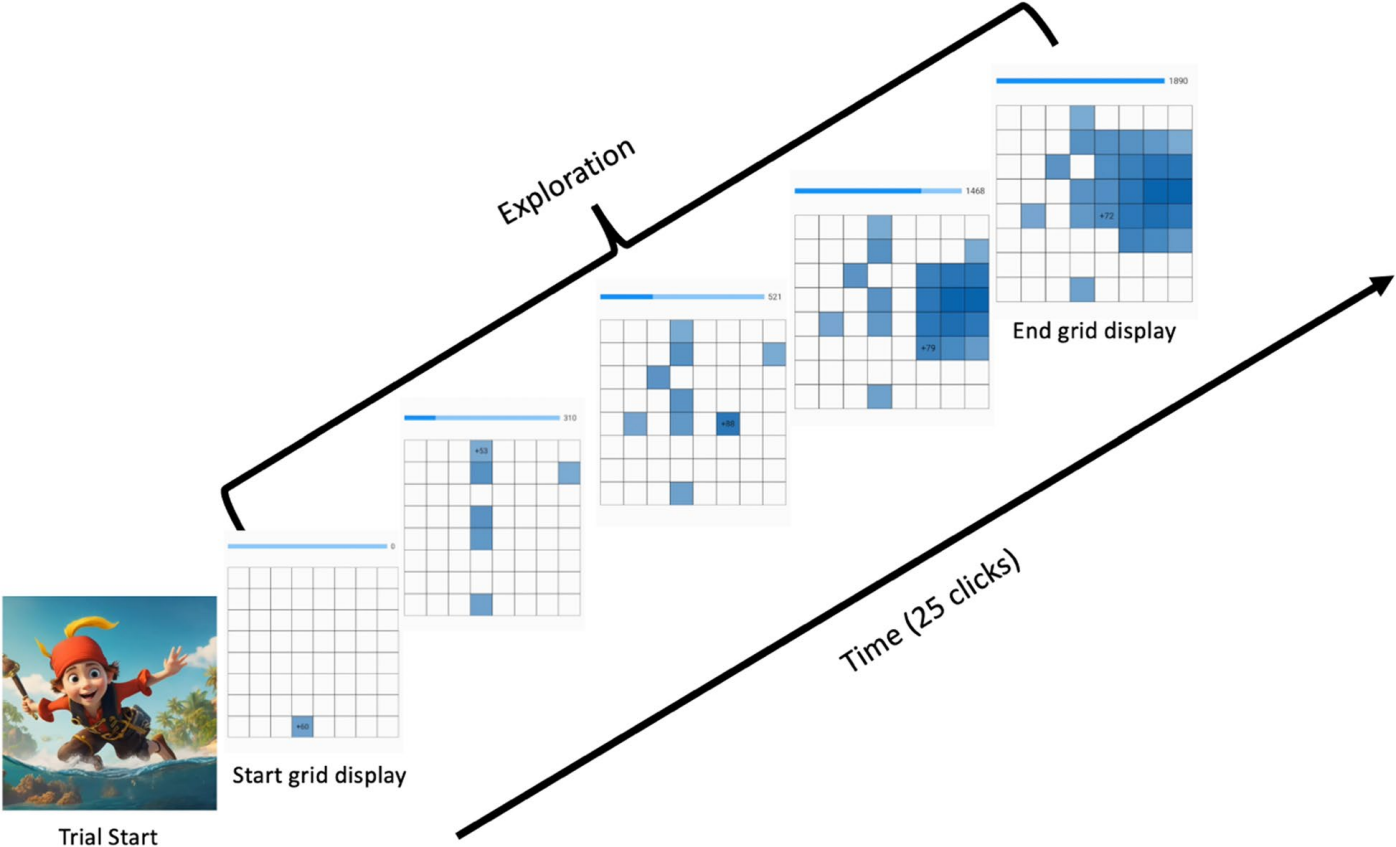
Exploration task procedure. Participants are presented with an 8-by-8 grid with 1 tile turned. The participants are instructed to click 25 times and try to find as many high treasures as possible. The grid will switch between a ‘smooth’ (high spatial correlations between the tiles) and a ‘rough’ (low spatial correlations between the tiles) grid at a random time during the session (after the initial 60 s and before the final 60 s). The type of grid that is presented first is randomised

*Exploration:* This task is based on Meder et al. [58]. Participants search for treasures in an 8-by-8 grid, see Fig. 6. Under each tile, a ‘treasure’ is hidden of differing values. A single ‘trial’ (reflecting a single treasure island grid) allows the participants to click 25 times on the tiles. Each click will reveal a number corresponding to the treasure (generally between 0 and 100), and a colour intensity corresponding to the value of the tile. Participants can either click on a new tile (with an unknown value), or click again on an already turned over tile (with a known value/reward), allowing an explicit distinction between explore and exploit behaviours. There are two different types of environments. In the ‘smooth’ environment, the value of the treasures are highly spatially correlated, making information about one tile’s treasure informative for nearby tiles. In the ‘rough’ environment, this correlation is low, resulting in seemingly randomly-dispersed treasures across the tiles of the grid. The environment will switch once during the task, at a random time during the task, but after the initial 60 s and before the final 60 s. The type of grid shown first is randomised. These environments favour different levels of exploration. Specifically, in the smooth environment, most treasures are obtained by exploiting knowledge about highly-rewarding tiles and only exploring nearby tiles. In the rough environment, on the other hand, large treasures could be obtained by exploring far tiles, favouring a high level of exploration in comparison to smooth environments, even when the number of clicks is almost used up [58]. Participants receive points for accuracy but not speed, because time pressure could have an impact on what strategy children use.

**Fig. 7:**
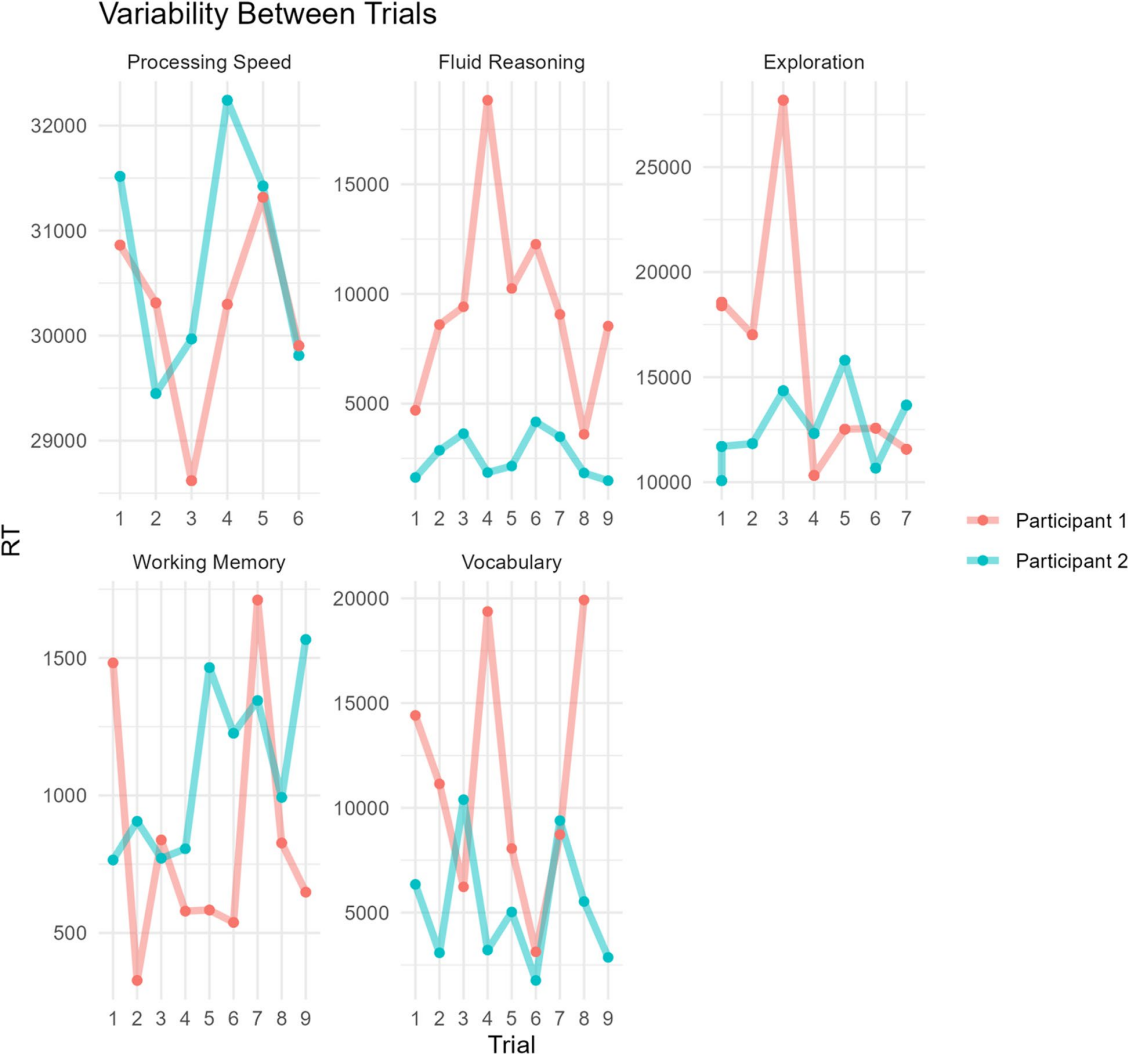
Example reaction time data for the initial trials of all cognitive tasks

For all five tasks we record performance (binary or continuous/press location) and speed (response time). Ambient background noise (decibels, not identifiable sounds) is measured at the start (before the first task starts) and the end (after finishing the final task) of each testing session. For a visual representation of the cognitive measures as part of the behavioural assessment, Fig. 7 illustrates sample response time data across the initial trials per task. Additional measures include 2 slide items before each block, on mood (smiley face to sad face) and sleep (alert face to sleepy face). After 10 sessions of performing the tasks, children are asked to rate the tasks from ‘least fun’ to ‘most fun’ to measure their motivation or enjoyment for the tasks. If consent has been given, academic results on national tests (e.g., Cito-scores) from the child are obtained through the school. Finally, in order to understand our sample, demographic measures of the child are collected through the informed consent form, such as age, sex, education grade/level, and whether they understand Dutch (which would be an exclusion criteria).

#### CODEC-MRI: imaging procedure

**Fig. 8:**
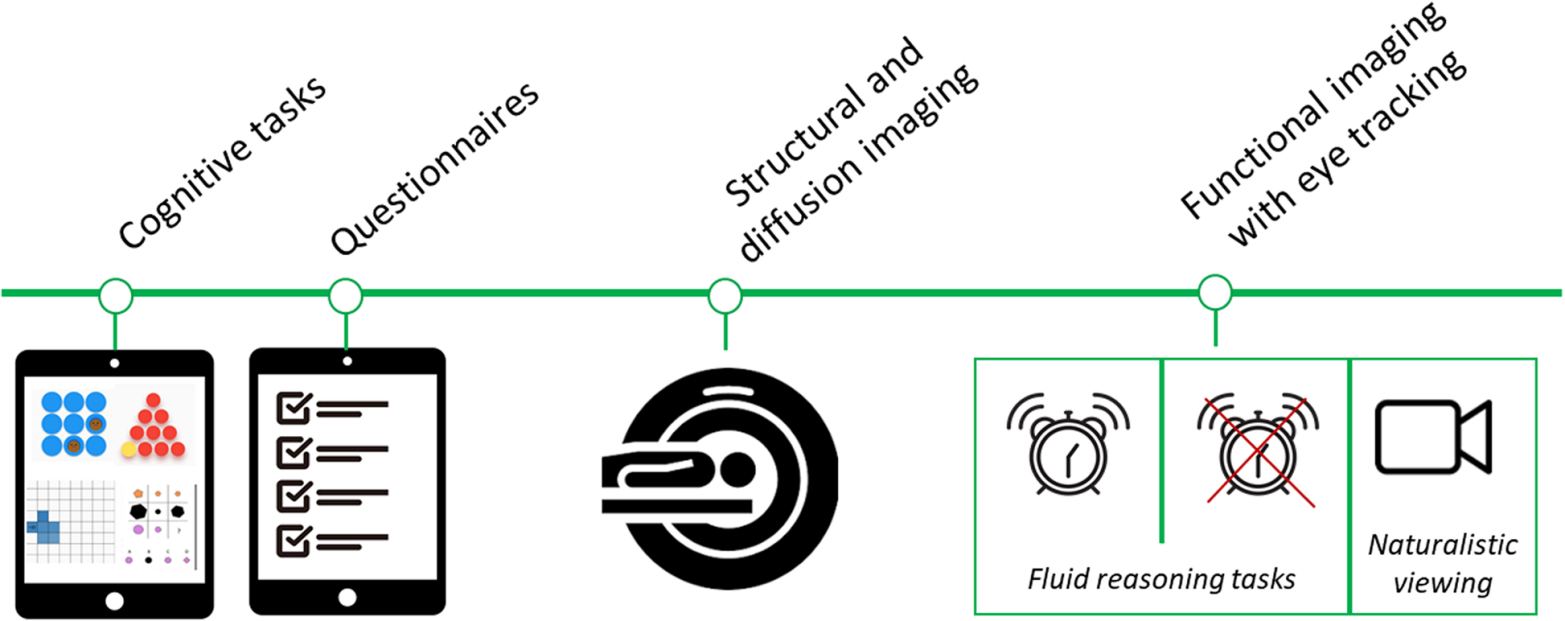
Deep phenotyping procedure. The deep phenotyping (the first and third year of the study) includes MRI (structural, functional, diffusion), and eye-tracking (pupillometry, gaze). Additionally, children and their parents perform cognitive tasks and a set of questionnaires

At the time of initial recruitment into the behavioural study, each participant (their guardian/parent) is asked whether they are also interested in participating in the imaging arm. If (after receiving and reading the imaging information packet) consent has been given for the child to take part in the imaging study, a visit to the Donders Institute is planned where the child receives an introduction and instructions on the day. Prior to the scanning session, a mock scan will be conducted to familiarise children with the scanner procedure and the cognitive tasks performed during the scanning, as well as to practise lying still. There are two blocks of the fluid reasoning task during the MRI session, one with a low time constraint and one with a high time constraint, to induce changes in strategy [107]. Figure 8 displays an overview of the imaging procedure.

The tasks are explained and practised in the mock scanner prior to the actual MRI scan. The order of the task blocks during the scan (high or low time constraints) are randomised.

Outside of the MRI scanner, children perform each of the five cognitive tasks for 3 min (15 min total). Children also respond to a short set of questionnaires (~15 min). While children are in the scanner, parents or guardians (if they also provide consent themselves) also respond to a set of questionnaires and perform the cognitive tasks on a tablet.

Details of the sequences of the MRI scan can be found in Table 1. The MRI scan consists of:

- MPRage and Sparse MP2rage: gold standard structural scans. MP2-Rage [108] allows for greater specificity of myelination, one of the core research questions.
- Naturalistic viewing fMRI: participants will watch a short video clip (~8 min), featuring a social scene from an age-appropriate, mainstream movie (Despicable Me).
- Task 1, fMRI (including eye-tracking/pupil dilation): a fluid reasoning task familiar to the children from the cognitive task battery, but with an additional condition of low or high time constraints.
- Task 2, fMRI (including eye-tracking/pupil dilation): fluid reasoning task with the opposite condition (low or high time constraints) compared to the first task block.
- Diffusion weighted imaging: this diffusion weighted sequence balances a realistic acquisition time with high quality imaging data.

**Table 1:** MRI sequences used in CODEC-MRI

Scan type	Sequence	TR (ms)	TE (ms)	Flip angle (°)	FOV (mm)	Voxel size (mm)	Other	Task
T1-weighted	MPRAGE	2300	3.03	8	256 × 256 × 192	1 × 1 × 1	GRAPPA: 2; TI: 1100 ms	None
T1-weighted	Sparse MP2RAGE	5000	2.88	Angle 1: 4 Angle 2: 5	240 × 256 × 224	1 × 1 × 1	TI 1: 700 (ms) TI 2: 2500 (ms)	None
Resting-state fMRI	EPI	1500	TE 1: 12.40 TE 2: 34.30 TE 3: 56.20	75	210 x 210 x 128	2.5 × 2.5x2.5	GRAPPA: 2; Volumes (N): 350; Slices: 51; Slice thickness: 2.5 (mm)	Movie watching
Task fMRI -Matrix reasoning task	EPI	1500	TE 1: 12.40 TE 2: 34.30 TE 3: 56.20	75	210 x 210 x 128	2.5 × 2.5x2.5	GRAPPA: 2; Volumes (N): 350; Slices: 51; Slice thickness: 2.5 (mm);	Visual stimuli and manual response
Field Map – Magnitude/Phase	PE-GRE	370	TE 1: 2.59 TE 2: 5.05	60	210 × 210 × 128	2.5 × 2.5x2.5	Volumes (N): 1; Slices: 51; Slice thickness: 2.5 (mm);	None
Diffusion-weighted (b = 0, b = 2000)	EPSE	3000	93	90	212 × 212 × 138	2 × 2 × 2	GRAPPA: 2; directions: 85; slices: 69 (axial); averages: 1; Bandwith: 1814 Hz/Px	None
Diffusion-weighted inversion (b = 0)	EPSE	3000	93	90	212 × 212 × 138	2 × 2 × 2	GRAPPA: 2; directions: 30; slices: 69 (axial); averages: 1; Bandwith: 1814 Hz/Px	None

*TR* Repetition time, *TE* Echo time, *TI* Inversion time, *FOV* Field of view, *EPI* T2*-weighted gradient echo planar image, *PE-GRE* Phase-encoded gradient echo

#### Imaging assessment

Before the imaging session in the scanner, children perform each of the five original cognitive tasks as used in the behavioural part for 3 min (15 min) and the child participants finish a set of tailored questionnaires and tasks. These are:

- *A digital mood scale:* a smiley question that assesses the child’s mood. The smiley changes along with the slider and ranges from an unhappy or sad smiley to a happy or joyful smiley. The number indicated by the slider ranges from 0 (unhappy smiley) to 100 (happy smiley).
- *A digital sleep scale*: a question that assesses the child’s sleep the evening before the session. The icon ranges from a bad night’s sleep (0) to a good night’s sleep (100).
- *Highly Sensitive Child scale – short form:* the HSC is a 12 item self-report scale (from 1-strongly disagree to 7-strongly agree) measuring environmental sensitivity. This scale has good internal consistency in children as young as 8 years ([109], for the Dutch version see [110]).
- *Mind Excessively Wandering Scale*: The MEWS is a 12 item self-report measure rating items on a scale from 0 (not at all or rarely) to 3 (nearly all of the time or constantly). The MEWS has an acceptable internal consistency in both adults and children from 8–13 years [111].
- *Alternative uses tasks:* children are asked to produce as many alternative uses for five physical objects (cap/hat, pen, cloth hanger, spatula, and a towel) for 2 min per object, based on Van Dijk et al. [112]. The experimenter reports the responses on a tablet, either by indicating one of the responses identified as an unoriginal answer in the study by Van Dijk et al. [112] or by writing out the answer. Fluency, flexibility, and originality are calculated.

During the imaging session, two blocks of fluid reasoning (2*8,5 min each) are performed by the child, one with low time constraints (30 s) and one with high time constraints (10 s). The time constraints are known to induce different task strategies which is a key variable of interest [66]. During the two task blocks, we record response time, accuracy, gaze direction, and pupil dilation (through the Eyelink 1000 Plus eye-tracking system). This requires a brief period of calibration (2 min) at the start of each fMRI task block.

Additionally, accompanying parents who agree perform a cognitive task battery identical to the one used in the behavioural arm for children and respond to a set of questionnaires about their child at each session. The set of questionnaires consists of:

- *Socio-demographic questionnaire:* a 15-item questionnaire assesses the child’s age, sex, handedness, grade, and the first and second language as well as the parents’ postal code, highest level of education attained, and job type and status.
- *Strengths and Difficulties Questionnaire*: the SDQ is a 25 item scale rating items from 0–2 (0-not at all/only a little, 1-quite a lot, 2-a great deal) providing a total difficulty score and five subscales consisting of five items each (hyperactivity, conduct problems, peer problems, emotional symptoms and prosocial). The parent-report scale has acceptable internal consistency [113].
- *MEWS:* the 12 item self-report scale assesses mind wandering and has good internal consistency in adults [114].
- *Behaviour Rating Inventory of Executive Function 2*: the BRIEF-2 is a questionnaire completed by parents to measure a child’s daily behaviour related to executive functions with good internal consistency [115].

### Subject retention strategies

In the behavioural arm, participation of the children is encouraged through the use of engaging, fun tasks and the potential to win points by doing the tasks. After the first ‘burst’ week, children receive a small soft brain as a reminder of their participation in the study. Teachers’ efforts are compensated by the option of up to three teaching packs (‘How to become a good researcher’, ‘Understanding the senses’, or ‘Understanding the brain’) which can be either delivered by teachers or by our team members as preferred. These teaching packs have been created by team members with prior experience in creating interactive teaching packs aimed at primary school children and with input from ‘Het Wetenschapsknooppunt’ [116] at Radboud University. An example and introduction can be found on the CODEC website: https://www.codecstudie.nl.

In the imaging arm, participation is made as enjoyable as possible for children, by means of child-friendly accents to the scanner (i.e., an age appropriate space decor). Participants receive compensation in the form of two tickets to the zoo after the first MRI session and a commemorative ‘certificate’. They have the possibility to receive a small 3D printed version of their brain after the second MRI session, which is given to the parents. Additionally, participants have the possibility to receive a picture of their brain after each scan.

With respect to the longitudinal element of the study, once a year we post a newsletter on the website with CODEC news, games for the children, and results of the studies. A paper copy of the newsletter is given to the teachers of participating classrooms. In addition, parents see a reminder when the next testing session will be planned at school or at home. Additional news for parents and teachers is provided through the CODEC website, https://www.codecstudie.nl.

### Quality management

All study procedures are being performed in participating schools or in home settings and some (CODEC-MRI) are performed at the Donders Institute for Brain, Cognition and Behaviour. The study team is extensively trained and certified to perform the various stages of the protocol. Standardised operating procedures (SOPs) are written documents detailing all procedures and what to do in case of adverse events or incidental findings, based on RadboudUMC best practices. A monitoring plan, based on internal policies and approved by the Radboud University Medical Centre’s Executive Board, has been developed to ensure a timely identification of deviations of the protocol or SOPs resulting in a decrease in the data quality. The monitor verifies that the rights and well-being of the subjects are protected and that the study is conducted in compliance with the approved protocol by checking key elements of the study (e.g., informed consent, inclusion procedure, subject source documentation). In the case where quality issues emerge, the study protocol and SOPs are modified accordingly and followed-up by the monitor.

### Data management and protection of subject privacy

Behavioural data collection through the m-Path app is managed as follows: the data is collected on tablets through the creators and administrators of the cognitive tasks, m-Path. The m-Path servers are located in Leuven and Heverlee, Belgium and are operated and secured by the KULeuven. Entrance to the servers is limited to the personnel maintaining the infrastructure. All data on the m-Path app (e.g., questions and answers, trial accuracy and reaction times) are stored in a protected folder on the tablets. This folder cannot be accessed by other apps. All the responses given by the participants are sent to the m-Path server through a secure HTTPS connection when the internet connection is (re)established. Data stored on m-Path servers are periodically sent to the Donders infrastructure. All communications between the m-Path app and the Donders Institute servers are executed using a secure HTTPS connection.

All data obtained during the imaging sessions is collected and stored at the Donders Centre for Cognitive Neuroimaging (DCCN) following gold-standard storing and back-up procedures. The Donders Institute has established a data management infrastructure to which all relevant data is sent. The data are stored online in a central storage space during data collection. Restricted access to study data on the central storage system is implemented by means of specific Unix user groups. Participant metadata is stored in password-protected CastorEDC databases. For individual electronic case report forms, we use the GCP-certified electronic data capture and management system: CastorEDC [117]. This data is entered directly into Castor and stored in a pseudonymised manner. Processing of all data on personal computers is protected by a site firewall. For data processing, raw data is uploaded to network attached storage that, in compliance with institutional guidelines, only researchers directly involved in the processing of data from the specific project can access. This is also applied to the imaging data. Our data is processed using the Donders Institute High-Performance Computing system [118] which provides the safest and most efficient use of in-house computational resources. To ensure data availability, a second copy of the data is stored on the Radboud Research data repository (https://data.donders.ru.nl) located on the Radboud University campus.

The investigator ensures that the subject’s anonymity is maintained. In all documents, subjects are identified by an identification code. The investigator keeps two separate Subject Identification Code Lists, which match (a) anonymous, memorable login codes (e.g., bluegiraffe12) with the subjects’ names and (b) anonymous login codes (e.g., bluegiraffe12) to the anonymous identification code (e.g., ID125). Participating parents receive an identification code and a memorable login code related to their child’s code (e.g., bluegiraffe12_M1/ ID125_M1). These documents are maintained in a secure password-protected location according to best practice.

After completion of the study, data is backed-up on password-protected and encrypted hard drives. Personal identifiers are stored separately from other metadata in a document safeguarded by the principal investigator. The investigator archives other essential documents in a study Master file. Handling of personal data complies with the Dutch Personal Data Protection Act, as well as the EU privacy laws. Following publication, raw and processed data are archived for scientific integrity. Personal data is kept separately from the experimental data acquired.

### Statistical analyses

CODEC provides an extremely rich longitudinal dataset which will be made available to the community through a managed access protocol. The CODEC dataset will generate a large number of potential questions, novel approaches and challenges. However, CODEC also sets out to answer a set of key preregistered hypotheses. Below we outline the general analytic framework, as well as 23 key preregistered predictions and analyses (https://osf.io/jzu6n/), all written prior to the first data collection.

#### Quantitative framework

To quantify variability, a wide range of measures are available. However, some simple summary metrics such as iSD (individual standard deviations) or ICV (coefficient of variation) do not take autoregressive structures or global change into account [119] and tend to ignore measurement error inherent in variability [120]. To overcome this challenge, we use a relatively new, flexible, integrative mathematical framework of Dynamic Structural Equation Modelling (DSEM) [121–123], allowing us to simultaneously estimate all components of the sequence of task trials. Moreover, each of the four key parameters (mean, trend, inertia and residual variability) can be estimated as a random effect (i.e., allowing for variations between individuals) within a multilevel Structural Equation Modelling (SEM) framework. Fitting a DSEM to the planned ‘burst’ measurement therefore results in a rich set of variability estimates across individuals, tasks and temporal resolutions.

#### Key hypotheses

**Aim (1) reliably measure individual differences in cognitive variability across timescales and tasks:** to overcome previous challenges in reliably measuring variability, we first use DSEM to estimate individual differences in variability across task domains (between the various tasks we assess), and across levels of temporal resolution (from trial-to-trial, across sessions within days, from day-to-day and from year-to-year). In these DSEM models we simultaneously estimate the mean, trend, autoregression, and residual standard deviation (see [25, 68], or [7] for more details). Moreover, each of these parameters is estimated as a random effect (i.e., as varying between individuals) and compared to a model with fixed effect parameters (i.e., assumed constant between individuals). One central expectation is that within person fluctuations are not (solely) measurement error, which we would expect to be similar across individuals. Instead, we hypothesise that within-person variability reliably differs between individuals for a given dataset with suitable temporal resolution. As such, our first hypothesis is that

- *Hypothesis 1.1: A DSEM with a parameter estimating the variance of subject-specific deviations in trial-to-trial variability will be preferred over a model that omits it, across all tasks.*
- *Hypothesis 1.2. A DSEM with a random-effects structure that freely estimates variability in mean performance from day-to-day (or session-to-session) will be preferred, over a model that only estimates fluctuations at faster timescales.*

Second, we expect that individual differences in variability will be correlated across tasks, but more weakly so than individual differences in (average) performance. Moreover, we expect that these differences cannot be fully captured with a single latent factor [7]. As such, our second preregistered prediction is as follows

- *Hypothesis 1.3: After extracting individual-level estimates of variability, we will fit a single factor model of variability across the 5 tasks, separately for each timescale. This unidimensional model will not fit sufficiently well (as indexed by a significant chi-square and an RMSEA* > *0.05). This prediction will hold for each timescale (trials, sessions, days).*

**Aim (2) Underlying mechanisms:** in the imaging arm, we consequently use key predictors of interest in the deeply phenotyped subsample (*N* = 200) to test mechanistic hypotheses about the (hypothesised) causes of individual differences in variability. In terms of brain structure, we first test the known association between larger grey matter volume and better cognitive performance to replicate previous findings [124]. Then, we test the neural noise hypothesis, which suggests that better myelination of the brain is associated with less variability due to decreased signal loss [73].

- *Hypothesis 2.1: Children with larger grey matter volume will demonstrate better cognitive performance in the behavioural tasks.*
- *Hypothesis 2.2: Children with larger white matter volume (better myelination) will demonstrate higher BOLD signal variability (potentially reflecting lower neural noise).*
- *Hypothesis 2.3: Children with higher tract-based indirect measures of myelination (both in white matter tracts and in grey matter) between frontoparietal executive systems (specifically: the SLF, ATR, IFOF and Forceps Minor) will show less variability. This effect will manifest most strongly for complex tasks, as those are most dependent on multi-region information integration.*
- *Hypothesis 2.4: Myelination increases during childhood. Children with steeper increase in myelination (both in white matter tracts and in grey matter) will show sharper decrease in behavioural variability between year 1 (*~*8 years old) and year 3 (*~*10 years old).*

Optimal cognitive performance often involves finding a balance between exploring different strategies and exploiting successful ones. For instance, for a matrix reasoning task, individuals may use either elimination (ruling out response options) or constructive matching (building a prediction, and then examining whether it is among the available options, [125]). Using fMRI data, we will study whether differences in strategy use govern individual differences in variability, and whether we can identify neural networks associated with different strategies as a potential mechanism underlying trial-to-trial variability. While exploring different strategies may temporarily reduce performance, it could facilitate long-term improvement by promoting exploration and adaptation. We also use eye-tracking data to infer foci of attention and task strategies, and changes in pupil dilation during the fMRI task blocks as a proxy for fluctuations in underlying noradrenergic and dopaminergic processes.

- *Hypothesis 2.5: Individuals with greater variability (estimated with DSEM) will show a larger number of response strategies within the fluid reasoning task (estimated through eye tracker analysis, cf. *[66]*).*
- *Hypothesis 2.6: Greater fMRI resting state variability (as quantified by blood oxygenation level-dependent standard deviations) is associated with better, and less variable behavioural performance in children.*
- *Hypothesis 2.7: Different trial strategies, estimated using eye tracker analysis *[66]* will be associated with recruitment of neural networks that differ qualitatively (i.e., different centroid and negligible spatial overlap).*

Additionally, we study the role of psychological (e.g., mind wandering, sleep, mood) and environmental (e.g., socio-economic status (SES)) factors on between- and within-person differences in variability by using them as predictors in a (regularised) SEM.

- *Hypothesis 2.8: Individuals from lower SES backgrounds will show greater day-to-day variability.*
- *Hypothesis 2.9: Individuals with greater mind wandering tendency will show greater cognitive variability, especially at the trial-to-trial level.*
- *Hypothesis 2.10: Days or testing sessions with greater negative affect, poorer sleep, and/or more background noise will be days or testing sessions with greater variability as well as poorer mean performance.*
- *Hypothesis 2.11: Tasks ranked as less enjoyable (individually) will be tasks with greater variability as well as poorer mean performance (when adjusting for task difficulty).*

To understand variability within individuals, we have to consider not only unique within-subject predictors but also potential subgroup differences. Neubauer et al. [43], identified subgroups of different profiles within a population for whom mood had varying effects on variability (i.e., low mood leading to higher variability, high mood leading to higher variability, mood having little effect on variability, and a group where both low and high mood lead to higher variability). We will use mixture models to identify subgroups within the population, allowing for the coupling of variability with factors like mood, sleep, background noise, and motivation (task rank). Specific subgroups that will be identified due to interactions between these factors are mostly exploratory, as not enough research has investigated this question so far.

- *Hypothesis 2.12 (exploratory): The overall sample will consist of a mixture of distinguishable subgroups in the population with a subgroup having a negative impact of mood, sleep quality, background noise, and motivation (task ranking) on variability and a subgroup having no negative impact of mood, sleep, background noise, and motivation (task ranking) on variability.*

The parents or guardians of the subgroup taking part in the imaging study are also asked to perform the cognitive tasks on tablet at each session. These scores act as predicting variables in latent mixed models to explore possible hereditary correlations.

- *Hypothesis 2.13: Variability will be correlated between parents and children*

**Aim (3) Long-term consequences:** Our last class of predictions concerns long-term outcomes of individual differences in variability. Using latent growth curve models, we will study how variability and mean performance evolve over time, and how differences in variability are associated with faster, or slower, rates of cognitive development and the emergence of subclinical neurodevelopmental symptomatology (e.g. ADHD symptoms).

- *Hypothesis 3.1: Variability will decrease over developmental time. Specifically, a growth model with variability as observed scores will show a mean negative slope (less variability over time), as well as significant slope variance (individual differences in the rate of change over time). A basis model (which can capture a decelerating decrease) will outperform a linear model.*
- *Hypothesis 3.2: Changes in variability across tasks will show distinct developmental trajectories, as variability reflects task-specific expertise. Specifically, a multivariable latent growth curve model with freely estimated slope covariances will fit better than a simpler model with these covariances fixed to unity (i.e., a unidimensional factors-of-curves model), in line with distinct developmental trajectories.*
- *Hypothesis 3.3: Greater variability on simple tasks, and low temporal resolution (day-to-day), is associated with poorer long-term outcomes (shallower developmental slopes).*
- *Hypothesis 3.4: Great variability on complex tasks, but only at the trial-to-trial level, is associated with better long-term outcomes (steeper slopes).*
- *Hypothesis 3.5: Greater variability at baseline will be associated with stronger ADHD symptomatology at baseline, as well as more rapid increase in ADHD related symptomatology.*

A secondary long-term aim is to link individual differences in variability to longitudinal academic success (Cito-scores).

- *Hypothesis 3.6: Greater variability on simple tasks, and low temporal resolution (day-to-day), is associated with poorer long-term academic achievement (shallower developmental slopes).*
- *Hypothesis 3.7: Great variability on complex tasks, but only at the trial-to-trial level, is associated with better long-term academic outcomes (steeper slopes).*

### Sample size considerations

#### Behavioural arm

The number of subjects needed in DSEMs have previously been examined through a series of Monte Carlo simulations [126]. Focusing on univariate two-level autoregressive models, they demonstrate that the DSEM performs well across a range of scenarios. For example, the estimation stability gained from a larger number of subjects is greater than the benefits of a large number of trials. Crucially, they demonstrate that DSEM achieves a highly reliable estimate of individual differences in trial-to-trial cognitive variability with anywhere upwards of 15 trials, given 200 subjects (average estimate over 500 repetitions/true value = 0.88, [126]).

**Fig. 9:**
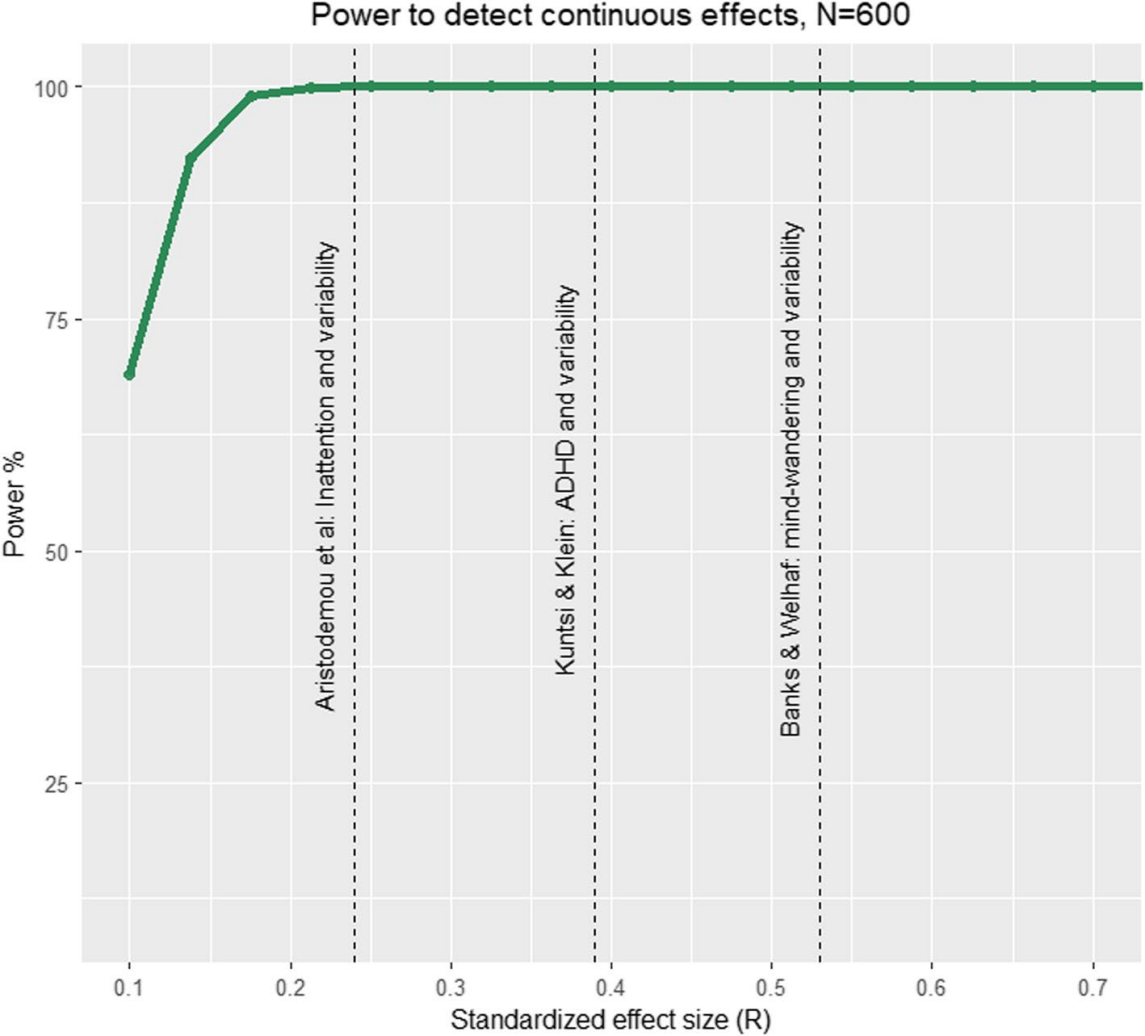
Power analysis based on our baseline sample size (600) and effects observed in our pilot analyses: A strong association between mind wandering and variability (*r* = -0.53), a moderate association between variability and ADHD (*r* = 0.39), and a small to moderate association between variability and inattention (median *r* = 0.24)

In terms of power to detect significant associations, the behavioural arm of the CODEC study is effectively at ceiling (see Fig. 9) for effect sizes we observe in previous literature, including the effect of mind wandering on variability (*r* = -0.53, 100% power; [52]), variability on ADHD symptoms (*r* = 0.39, 100%; [94]), and inattention on variability (*r* = 0.24, 100% power; [19]). For the full behavioural sample (*N* = 600), even small effect sizes (*r* = 0.11) can be detected with 80% power.

**Fig. 10:**
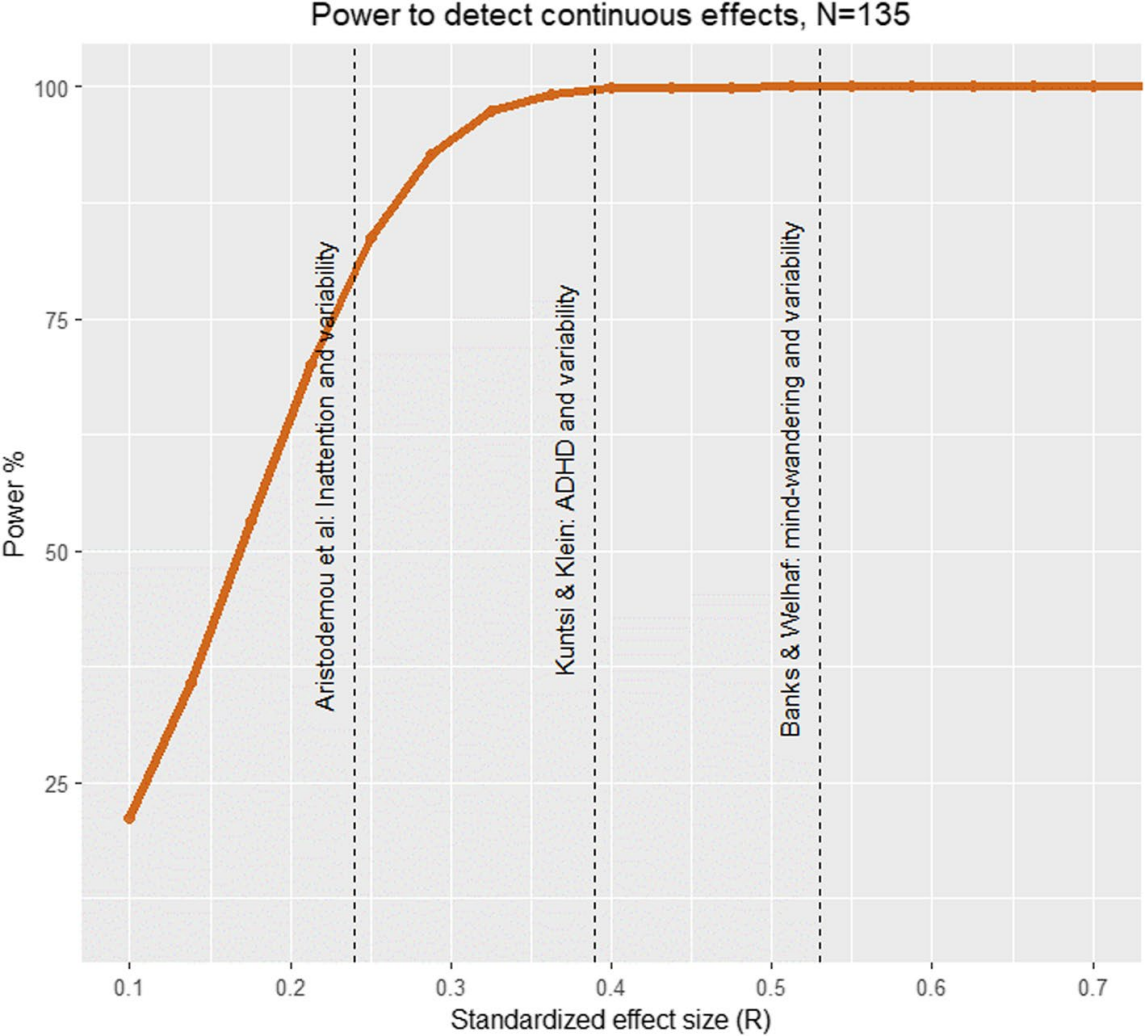
Power analysis based on the lowest sample size per subgroup (135) to detect effects observed in our pilot analyses at minimum 80% power

Our sample size of 600 children consists of children tested in schools and on an individual basis at home. By including participants that are recruited individually and tested at home, we add a new variable of a situational context. This enables us to test potential differences in cognitive variability between school and home environments. Factors such as systematic differences, the role of self-initiating sessions, the absence of peer influence, and variations in noise levels between both contexts are likely to have distinct impacts on cognitive variability. To ensure sufficient power for each group, we hope to recruit at least 135 participants tested at home, allowing us to both ensure precision of parameter estimation within each group (home and school based), as well as detect the hypothesised effect size group difference between the two with > 80% certainty (see Fig. 10).

Parallel to this, to understand the processes related to variability in children, our study considers various potential variables underlying cognitive variability (i.e., neural, psycho-social, cognitive, genetic). Our goal is to determine how each variable influences variability and to detect individual differences in this regard. Therefore, it is important not only to consider variables as unique predictors to variability but also to account for potential different subgroups of participants. Individuals are likely to belong to distinct classes or groups for whom variability is potentially more affected by certain variables, such as sleep or mood, while others may not exhibit the same impact. Consequently, we aim to explore different variability profiles among individuals, each characterised by unique underlying patterns. Therefore, our sample of 600 is divided based on the identified profiles. To ensure sufficient power for each group, we aim for the smallest subgroup size to be around 135 participants, allowing us to detect our expected effect sizes with 80% certainty (see Fig. 10). This enables us to investigate whether the overall sample can be considered a mixture of different distributions arising from distinguishable subgroups in the population with different underlying processes.

#### Imaging arm

**Fig. 11:**
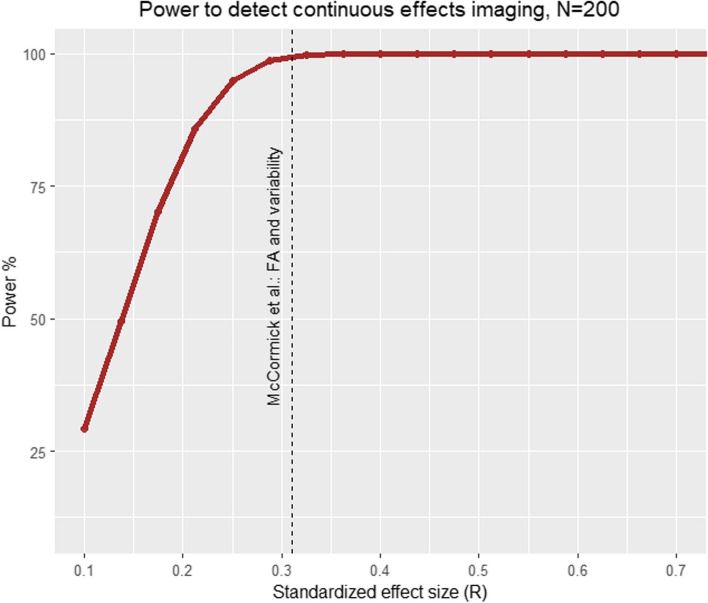
Power analysis to detect continuous effects in the imaging subsample of 200 participants

For the smaller imaging subsample the CODEC study has > 80% power to detect modest (0.2) associations and at > 99% power to detect moderate (*r* = 0.3) effects, see Fig. 11. This is sufficient to detect the expected moderate effects in line with previous findings (between neural data - Fractional Anisotropy (FA) and variability = 0.34; [68]).

## Discussion

Children’s cognitive performance fluctuates across temporal resolutions, and these fluctuations are known as within-subject cognitive variability. Cognitive variability could act as a potential early predictor of neurodevelopmental disorders [20, 21]. Despite the potential importance of cognitive variability, multiple factors including a lack of appropriate quantitative techniques and the unavailability of sufficiently rich datasets (including estimates across various tasks and temporal resolutions), cognitive variability, its underlying mechanisms and long-term implications are relatively neglected in the current literature.

The CODEC study addresses these remaining gaps by combining an accelerated longitudinal design including a behavioural ‘burst’ measurement (3 times per day, 5 days per week) and deep phenotyping, including fMRI, eye-tracking and a set of questionnaires, with advanced statistical methodologies. 600 children (age 7–12 at first measurement) are followed for up to 3 years as they undergo rapid cognitive development. Each year consists of a ‘burst’ week of experience sampling assessments of five cognitive domains (reasoning, working memory, processing speed, vocabulary, exploration), sleep quality, mood, and background noise, conducted both in classrooms and home settings. Academic outcomes and task preference are also collected once a year. A subset of 200 children of the CODEC participants take part in the deep phenotyping sessions (CODEC-MRI), once at the start of the study, in year 1, and once at the end in year 3. In addition to the cognitive battery, the deep phenotyping session consists of structural and functional MRI, eye-tracking, parental measurements (on the cognitive battery) and questionnaire-based demographic and psychosocial measures as reported by parents and children. Analysing the data through Dynamic Structural Equation Modelling, allows us to simultaneously capture variability and the multilevel structure of trials nested in sessions, days, children and classrooms. In doing so, we aim to pave the way to a better understanding of cognitive variability and its implications, providing insight into the mechanisms and consequences.

In line with the open-data principles, the data generated by the CODEC study will be made openly available for scientific reuse to be shared with fellow researchers when data can be shared securely. There is no cost attached to the reuse of the data. Data sharing is done using the Radboud research data repository [127] in accordance with institutional, Radboud University, and EU guidelines. We share only anonymised behavioural data, and anonymised (derived) (f)MRI data.

The findings of this study lead to insight in three main areas: (1) the reliable measurement of individual differences in variability, (2) the underlying predictors and potential causes, and (3) the long-term trajectories and consequences of cognitive fluctuations. In conclusion, the CODEC study aims to provide valuable insights into the mechanisms, consequences, and potential interventions related to cognitive variability, ultimately contributing to more equitable assessments and improved support for young individuals with variable cognitive performance.

## Data Availability

The anonymised datasets generated during this study will be made openly available to researchers for scientific reuse that is not limited to the research project for which the data was initially collected, after completion of data collection and cleaning. There will be no cost for reusing the data. Data may be shared with researchers/institutions in countries within the EU where the AVG (Algemene Verordening Gegevensbescherming) applies. We will only share data with researchers/institutions in countries outside the EU, where the AVG does not apply, if we can ensure that the data can be shared securely and protected appropriately in accordance with the AVG. When this is not possible, we are not able to share the data outside of the EU. Participants have provided consent for the reuse of their anonymised data for scientific purposes. Data sharing is done using the Radboud research data repository (http://data.donders.ru.nl) in accordance with institutional, Radboud University, and EU guidelines. We will share only anonymised behavioural data, and anonymised (derived) (f)MRI data through a managed access format (users will submit a brief research proposal and which variables are needed to conduct the relevant analyses). Raw (f)MRI files cannot be not shared externally. Collected data for this project is stored in digitised data archives, maintained by the IT department of the Donders Institute.

## Abbreviations

ADHD: Attention-deficit/hyperactivity disorder
ASD: Autism spectrum disorder
BOLD: Blood oxygen level-dependent
BRIEF-2: Behaviour Rating Inventory of Executive Function 2
CODEC: COgnitive Dynamics in Early Childhood
DCCN: Donders Centre for Cognitive Neuroimaging
DSEM: Dynamic Structural Equation Modelling
FA: Fractional anisotropy
fMRI: Functional magnetic resonance imaging
HSC: Highly Sensitive Child scale
ICV: Coefficient of variation
iSD: Individual standard deviations
MaRs-IB: Matrix Reasoning Item Bank
MEWS: Mind Excessively Wandering Scale
SDQ: Strengths and Difficulties Questionnaire
SEM: Structural Equation Modelling
SES: Socio-economic status
SOPs: Standardised operating procedures

## Footnotes

1 The age of the children for the behavioural arm is not strictly set at 8 years to avoid children in the same classroom (when they turn 8 later in the year) to not be allowed to participate and to allow for a large enough sample size. 7-year-old children are allowed to take part in the imaging arm upon turning 8.
